# Human hepatocyte depletion in the presence of HIV-1 infection in dual reconstituted humanized mice

**DOI:** 10.1242/bio.029785

**Published:** 2018-01-22

**Authors:** Raghubendra Singh Dagur, Weimin Wang, Yan Cheng, Edward Makarov, Murali Ganesan, Hiroshi Suemizu, Catherine L. Gebhart, Santhi Gorantla, Natalia Osna, Larisa Y. Poluektova

**Affiliations:** 1 Department of Pharmacology and Experimental Neuroscience, University of Nebraska Medical Center, Omaha, NE 68198, USA; 2 Research Service, Veterans Affairs Nebraska-Western Iowa Health Care System, Omaha, NE 68198, USA; 3 Department of Internal Medicine, University of Nebraska Medical Center, Omaha, NE 68198, USA; 4 Laboratory Animal Research Department, Central Institute for Experimental Animals, 3-25-12 Tonomachi, Kawasaki, Kawasaki 210-0821, Japan; 5 Molecular Diagnostics Laboratory, Department of Pathology and Microbiology, University of Nebraska Medical Center, Omaha, NE 68198, USA

**Keywords:** Humanized mouse model, Human immune system, Liver reconstitution, Human immunodeficiency virus-1, Hepatocytes, Hematopoietic stem/progenitor cells, Toll-like receptors

## Abstract

Human immunodeficiency virus type 1 (HIV-1) infection impairs liver function, and liver diseases have become a leading cause of morbidity in infected patients. The immunopathology of liver damage caused by HIV-1 remains unclear. We used chimeric mice dually reconstituted with a human immune system and hepatocytes to address the relevance of the model to pathobiology questions related to human hepatocyte survival in the presence of systemic infection. TK-NOG males were transplanted with mismatched human hematopoietic stem/progenitor cells and hepatocytes, human albumin concentration and the presence of human immune cells in blood were monitored for hepatocytes and immune reconstitution, and mice were infected with HIV-1. HIV-1-infected animals showed a decline in human albumin concentration with a significant reduction in percentage of human hepatocytes compared to uninfected mice. The decrease in human albumin levels correlated with a decline in CD4^+^ cells in the liver and with an increase in HIV-1 viral load. HIV-1 infection elicited proinflammatory response in the immunological milieu of the liver in HIV-infected mice compared to uninfected animals, as determined by upregulation of IL23, CXCL10 and multiple toll-like receptor expression. The inflammatory reaction associated with HIV-1 infection *in vivo* could contribute to the depletion and dysfunction of hepatocytes. The dual reconstituted TK-NOG mouse model is a feasible platform to investigate hepatocyte-related HIV-1 immunopathogenesis.

This article has an associated First Person interview with the first author of the paper.

## Abstract (precis)

**Summary:** We describe a model that recapitulates multiple components of liver damage by HIV-1 infection as in humans, including reduced liver CD4+ cells, albumin levels, liver immune activation and human hepatocyte survival.

## INTRODUCTION

Liver injury remains an important contributor to morbidity and mortality among ∼1.2 million persons in the USA and nearly 37 million human immunodeficiency virus (HIV)-infected patients worldwide (Smith et al., 2010). HIV type 1 (HIV-1) causes damage of liver cells, i.e. hepatocytes (Hep), stellate cells and Kupffer cells; and direct and indirect inflammation-associated mechanisms were suggested and investigated *in vitro* (Crane et al., 2012; Kong et al., 2012). However, *in vitro* cell culture experiments are limited in various ways, and the results of human studies vary based on time of infection, co-infections with hepatitis viruses, opportunistic infections, antiretroviral drug treatment, and damage of gut immune barriers and associated immune activation (Sherman et al., 2015). More coherent information can be obtained *in vivo* using HIV-1-infected chimeric mice carrying human hemato-lymphoid tissue and human Hep to recapitulate HIV-1 immunopathogenesis. However, syngeneic Hep and human CD34^+^ hematopoietic stem/progenitor cell (HSPC) co-transplantation has limitations due to availability of matched cells and uncertainties. We sought to investigate how a mismatched model and possible alloreactivity would affect outcomes. To explore such mismatched experimental conditions, we created dual liver- and immune system-transplanted humanized mice and addressed the effects of HIV-1 on hepatocyte survival and function. In this experimental system, we used Hep derived from a single donor for transplantation into NOD/scid-IL2Rγ_c_^null^ (NOG) mice expressing the herpes simplex virus type 1 thymidine kinase (*TK*) transgene within the liver (TK-NOG) (Hasegawa et al., 2011). A human immune system was established by co-transplantation of human CD34^+^ HSPCs derived from eight single donors. Experimental animals were infected with macrophage-tropic CCR5 co-receptor using the HIV-1_ADA_ strain. Five weeks post-infection, we found a decreased concentration of human albumin (ALB) in the blood, which coincided with a reduced number of engrafted human Hep in the liver of dual reconstituted mice. It is noteworthy that we observed no changes in human ALB levels and preserved Hep in the uninfected mice transplanted with mismatched cells, which suggests that HIV-infection contributes to hepatocyte injury. To explore the processes that could be involved in Hep damage in dual reconstituted humanized mice, we evaluated the presence of infected cells in liver tissue as well as the pathomorphology associated with this observation. We also compared profiles of inflammation-associated gene transcripts in the livers of infected and uninfected dual humanized mice at the end-point of observation to explore liver-immune cell milieu. HIV-1-infection elicited significant upregulation of pattern-recognition receptors and inflammatory cytokines/chemokines *in vivo*. The depletion of human hepatocytes and the decline of human ALB levels *in vivo* could be results of HIV-1 infection of the liver-immune cell milieu and not related to mismatched transplantation of human HSPCs and hepatocytes.

## RESULTS

### HIV-1 infection reduces human ALB levels and hepatocyte population in dual reconstituted mice

**Fig. 1.**
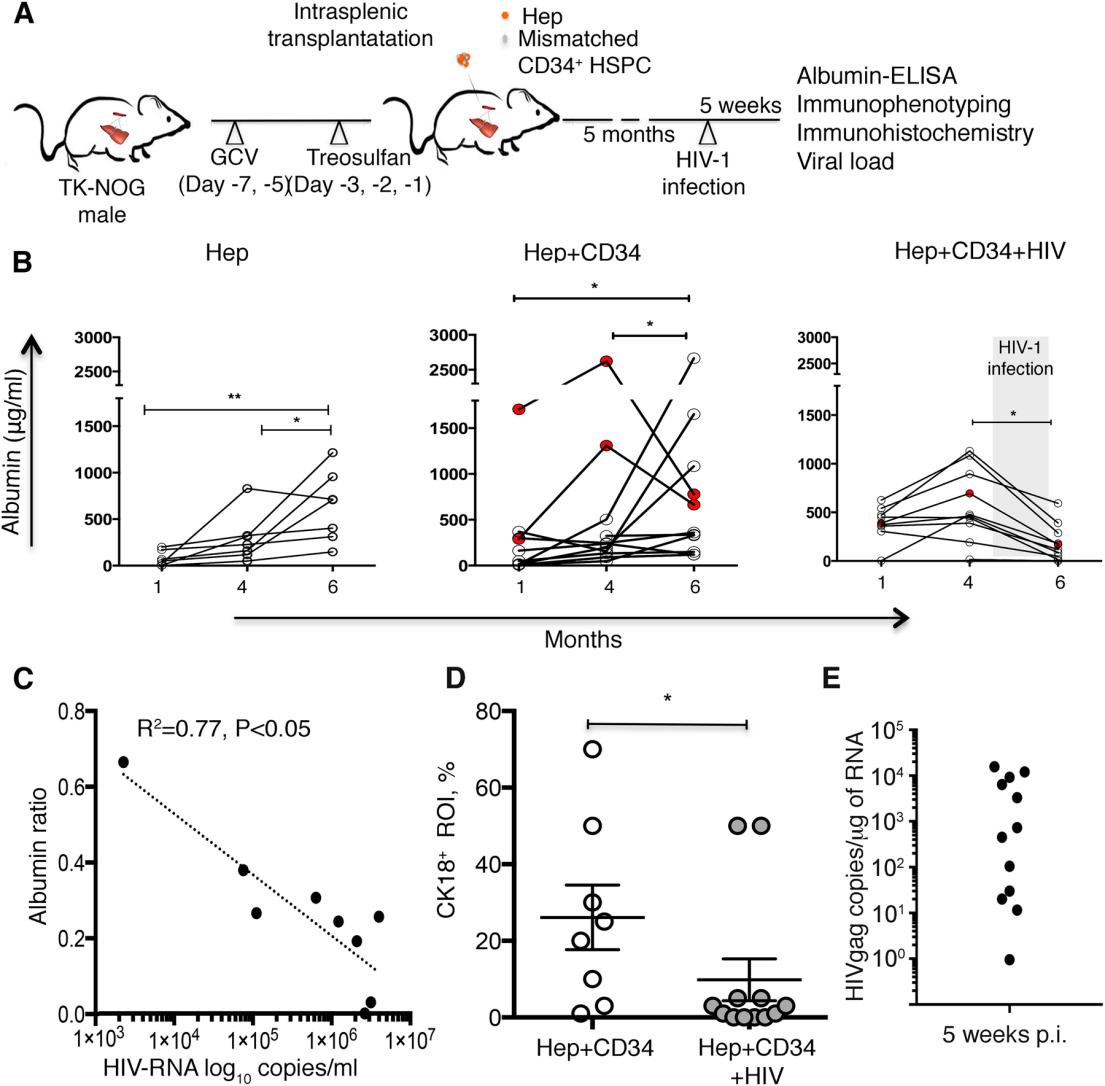
**HIV-1 infection affects human hepatocytes in dual liver and immune system humanized mice.** (A) Experimental design for dual reconstitution of humanized liver and immune system mice. TK-NOG males were intraperitoneally injected with GCV and treosulfan on the indicated days before intrasplenically transplantation with both Hep (orange, hexagons) and CD34^+^ HSPCs (gray circles). Levels of ALB and presence of immune cells were assessed to monitor liver and immune system reconstitution, respectively. Dual reconstituted mice at the age of ∼5 months were infected with HIV-1 and sacrificed 5 weeks post-infection to study HIV-1-associated liver pathogenesis. (B) ALB concentrations were monitored in mice transplanted with human Hep (*n*=7) reconstituted with both human CD34^+^ HSPCs and Hep without HIV-1 infection (Hep+CD34, *n*=11) and with HIV-1_ADA_ infection (Hep+CD34+HIV, *n*=10). Each symbol represents an individual mouse value. The shaded box indicates the HIV-1 infection period. *P<0.05 and **P<0.005 is within each group, by one-way ANOVA. Two uninfected dual-reconstituted animals reconstituted with CD34^+^ HSPCs from the donor ‘C’ experienced ALB reduction regardless of HIV-1 infection, so the ALB drop in HIV-infected mouse could be caused by something other than HIV-1 infection. These animals were excluded from ALB and correlation analyses (red circles) (see Table S1). (C) Graph shows the Pearson correlation of HIV-1 viral load (HIV-RNA log_10_ copies/ml) to ALB ratio [ratios of ALB concentration at 5 weeks post-infection and pre-infection ALB levels at 4 months after Hep transplantation (*n*=9, *P*<0.05, R^2^=0.77)]. (D) Liver sections obtained from dual humanized uninfected (Hep+CD34) (open circles, *n*=8) and HIV-1-infected (Hep+CD34+HIV) (closed circles, *n*=12) mice were stained with anti-human CK18 antibodies. Fields (3-5) of view under objective magnification 10× were analyzed and the area occupied by the CK18^+^ human cells was expressed as percentage in region of interest (ROI). Results represent the mean±s.e.m. as well as individual values. *P<0.05 between uninfected and HIV-infected mice, by one-way ANOVA. (E) Presence of HIV-infection 5 weeks post-infection (p.i.) of HIV-1 is shown on the *y*-axis by transcript copies of HIV*gag* in 1 μg of total RNA, by relative standard curve method in HIV-infected mice (*n*=12) using semi-nested PCR.

To assess the effect of HIV infection on the liver of dual reconstituted mice, we transplanted mice with human Hep from a single donor and human CD34^+^ HSPCs from single donors simultaneously to achieve double reconstitution with human liver cells and a hemato-lymphoid system. TK-NOG males were conditioned with ganciclovir (GCV) for mouse hepatocyte depletion and myeloablated with treosulfan for HSPC transplantation (Fig. 1A). Human ALB and the presence of human CD45^+^ cells in blood were evaluated to monitor the repopulation of mouse livers with Hep and reconstitution with human immune cells, respectively. At 1 month post-transplantation, levels of human ALB in mouse peripheral blood were higher in the groups co-transplanted with CD34^+^ cells, showing a median of 302 μg/ml (Hep+CD34 mice, range 3.63–626 μg/ml) compared to animals transplanted with Hep alone, reaching only a median of 51.1 μg/ml (range 1–171.7 μg/ml, *P*<0.05) (Fig. 1B). When humanization of liver and immune system establishment was evident (∼5 months of age), mice were infected with HIV-1_ADA_ 10^4^ tissue culture infectious doses 50 (TCID_50_) intraperitoneally, and euthanized at 5 weeks post-infection (Hep+CD34+HIV mice). We found that in HIV-infected mice, post-infection concentration of ALB was reduced to a median value of 118.8 μg/ml (range 0–593 μg/ml) from a pre-infection median value of 461 μg/ml (range 389-1131) within the group. By contrast, in uninfected, dual humanized mice and hepatocyte-transplanted mice, ALB levels increased to a median value of 336 μg/ml (range 117.6–2663 μg/ml) and 708 μg/ml (range 148.7–1215.7 μg/ml) post-sham infection from their median values of 196 μg/ml (range 48.4–503 μg/ml) and 227 μg/ml (range 50–828.5 μg/ml), respectively (Fig. 1B). The reduction of ALB concentration (ratio of ALB concentration at 5 weeks post-infection to pre-infection ALB levels at 4 months after Hep transplantation) correlated with peripheral viral load (R^2^=0.77, *P*<0.05) (Fig. 1C). Two uninfected mice and one HIV-infected dual humanized mouse transplanted with the donor ‘C’ CD34^+^ HSPCs showed decline in ALB levels; however, uninfected mice retained higher ALB (779.5 and 664 μg/ml), as compared to the HIV-infected mouse (169 μg/ml). These three animals were excluded from the ALB and correlation analysis (Table S1). Staining of humanized liver sections with human-specific anti-cytokeratin18 (CK18) antibodies and quantification of randomly selected areas on coded slides occupied by human Hep [expressed as percent of the region of interest (ROI)] revealed significant reduction (*P*<0.05) of CK18^+^ human Hep compared to uninfected mice (Fig. 1D). This indicates that we are dealing with actual depletion of human hepatocytes in the livers of HIV-infected mice. The presence of HIV-1 infection in liver tissue samples was assessed by reverse transcription polymerase chain reaction (RT-PCR) and showed variable levels (Fig. 1E). We observed a negative correlation between ALB levels and HIV*gag* copies in the liver of HIV-infected dual humanized mice by Spearman's correlation; however, the effect was not statistically significant (*r*=−0.71, *P*>0.05).

### HIV-1 infection depletes human CD4^+^ cells in peripheral blood, spleen and liver, and is associated with a decrease in human ALB levels

**Fig. 2.**
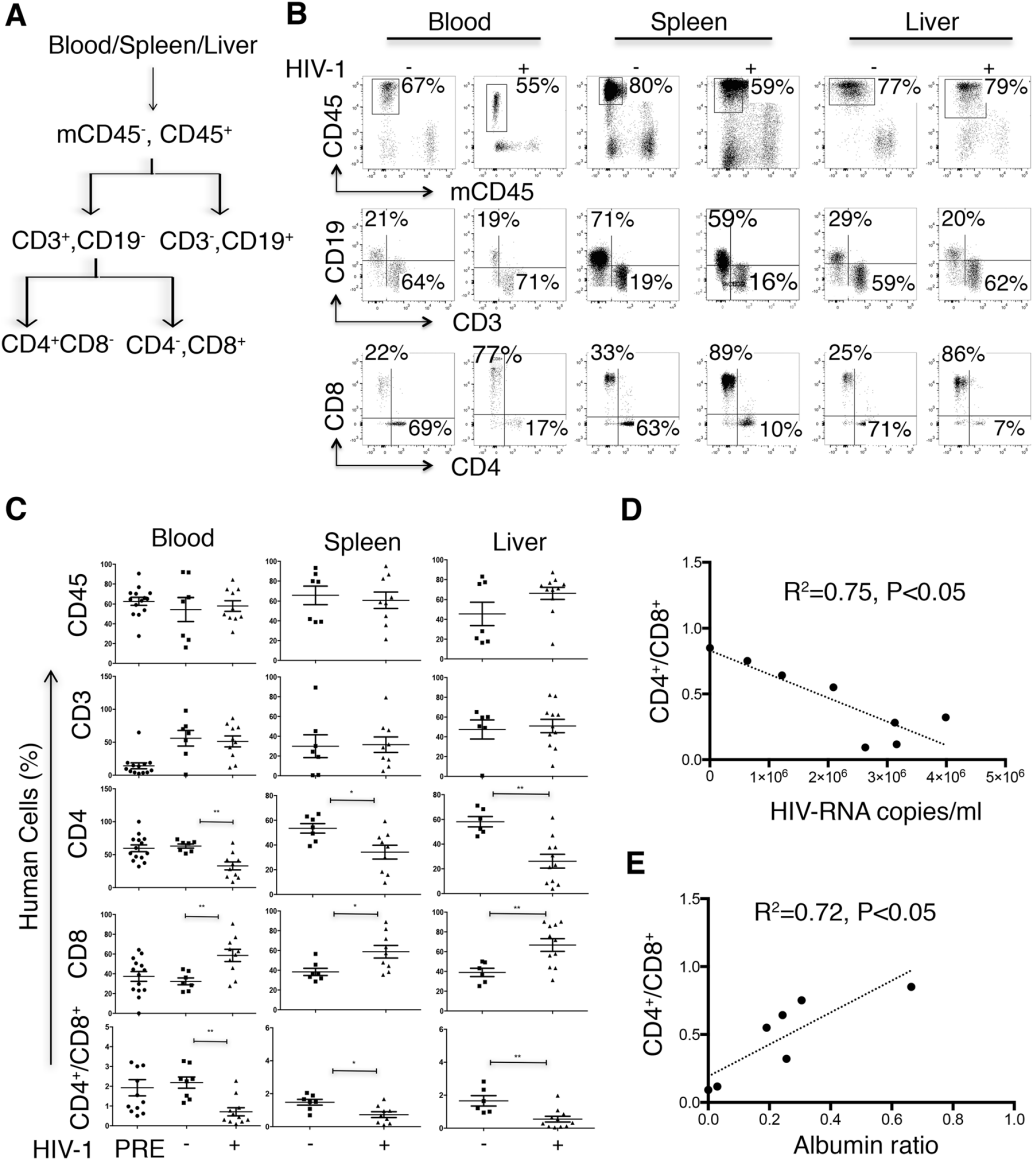
**HIV-1 infection affects immune cell distribution in blood, spleen and liver tissues.** (A) Gating strategy for analyzing frequency human cells distribution for blood, spleen and liver tissues. The results are expressed as percentages of the total number of gated lymphocytes. The gating strategy was human CD45→CD3/CD19, CD3→CD4/CD8. (B) Representative dot plots for uninfected (HIV-1−, *n=*7) and HIV-infected (HIV-1+, *n=*9) dual humanized mice showing human immune cell distribution in the blood, spleen and liver of mice. mCD45 indicates mouse specific CD45^+^ cells. Numbers indicate the percentage of gated cells. (C) Frequency of human cells in peripheral blood, spleen and liver of dual reconstituted uninfected (squares, HIV-1−, *n*=7) and HIV-1 infected (triangles, HIV-1+; *n*=9) mice. Pre-infection (PRE) in blood panel indicates the frequency of human cells prior to HIV-1 infection. Results represent the mean±s.e.m. as well as individual values. *P<0.05, and **P<0.005 by one-way ANOVA test between HIV-infected and uninfected mice. (D) Pearson correlation of CD4^+^/CD8^+^ ratio (ratios of CD4^+^ to CD8^+^ cells at 5 weeks post-infection) with HIV-1 RNA copies/ml in peripheral blood (*n*=8, R^2^=0.75, *P*<0.05) and (E) with albumin ALB (ratios of ALB concentration at 5-weeks post-infection to pre-infection ALB levels at 4 months after Hep transplantation) (*n*=7, R^2^=0.72, *P*<0.05).

**Fig. 3.**
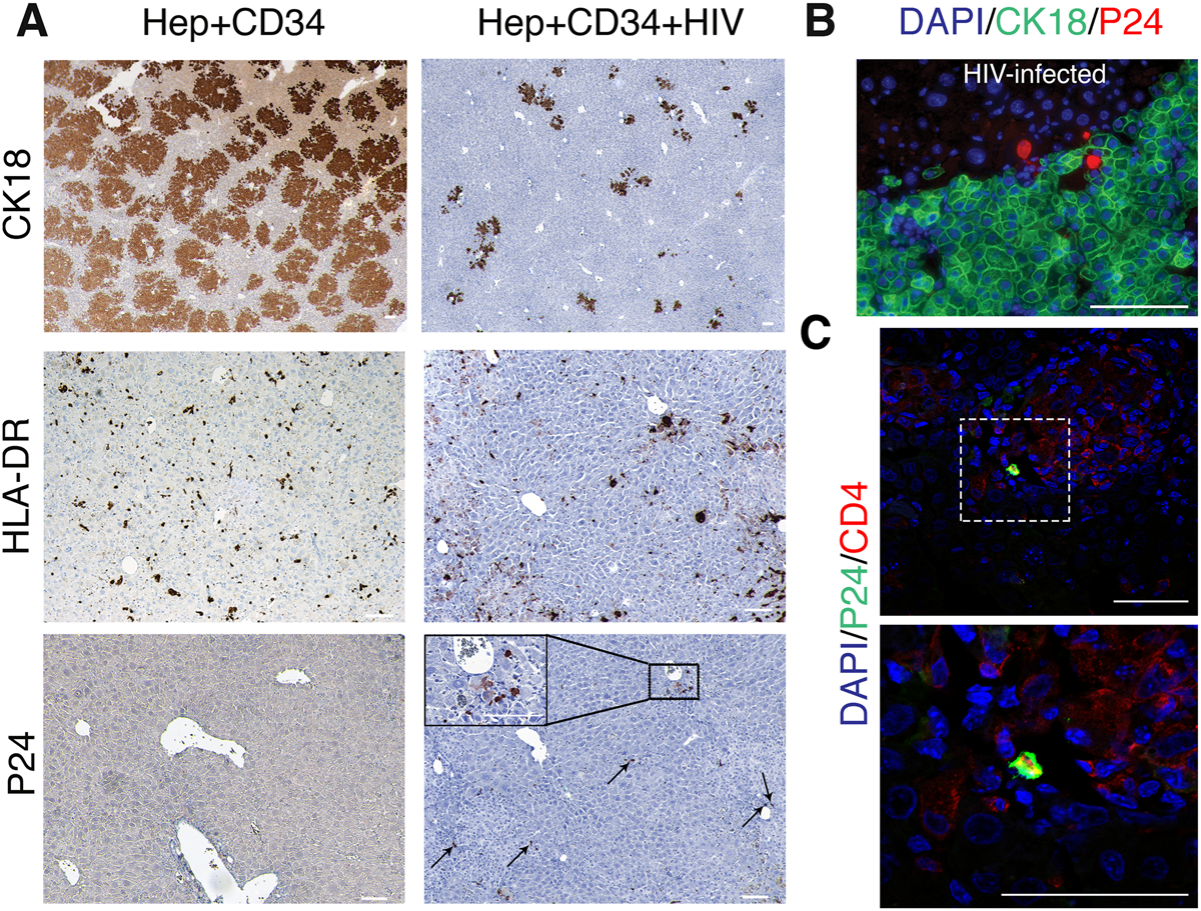
**Liver pathomorphology in HIV-1-infected dual reconstituted mice.** (A) Liver tissues of uninfected and HIV-1-infected TK-NOG mice were fixed, paraffin embedded, and 5-μm sections were stained for CK18 human HLA-DR, p24 and cytokeratin-18 (CK18), as indicated. Human cells positive for p24 staining were observed around the portal vein (arrows) and in a magnified view (inset) of the boxed area. Cells positive for HIV-1 p24 antigen are lymphocytes in morphology (inset). Scale bars: 100 μm. (B) Immunofluorescence staining for human p24^+^ (red) and CK18^+^ (green) cells in uninfected and HIV-infected dual humanized mice (top panels). p24^+^ (red) cells have the morphology of lymphocytes. Images were acquired on a Nikon Eclipse 55i using a Nuance multispectral imaging system. Scale bar: 100 μm. (C) Immunofluorescence image showing a subset of CD4^+^ lymphocytes (red) positive for p24 antigen (green). Liver sections were counterstained with DAPI (blue). The bottom image is a magnified view of the boxed area from the top image. Images were captured on Zeiss 710 confocal laser scanning microscope. Scale bars: 50 μm.

To investigate the effect of HIV-1 infection on the human immune system, we analyzed T cells (CD4^+^ and CD8^+^) in the peripheral blood, spleen and liver (Fig. 2). Lymphocytes and single cells were gated, as shown in Fig. S1. Before infection, mice were bled, and the presence of human immune cells was then tested in the blood. All the selected mice for infection and uninfected groups efficiently developed a human immune system, as shown by the presence of human CD45^+^ cells (62±4%), and T cells including CD4^+^ (mean±s.e.m. 60±5%) and CD8^+^ (38±5%) cells (Fig. 2C, left panels, PRE). A further gating strategy for assessing the frequency of human cells in blood, spleen and liver is illustrated in Fig. 2A. In all analyzed compartments, a significant decrease in the frequency of human CD4^+^ cells in blood (33±6%), liver (26±6%) and spleen (34±6%) were observed in HIV-infected compared to uninfected mice blood (63±3%), liver (58±4%) and spleen (53±4%) (Fig. 3B,C). We observed a significant increase in the percentage of human CD8^+^ cells and a subsequent decrease in the CD4^+^ to CD8^+^ ratio in the liver, spleen and blood of HIV-infected mice. We were interested to see a significant correlation between an increased viral load on the one hand, and a decreased liver CD4:CD8 ratio (R^2^=0.75, *P*<0.05) on the other (Fig. 2D), as well as an inverse correlation between a decreased liver CD4:CD8 ratio and human ALB concentration (R^2^=0.72, *P*<0.05) (Fig. 2E). However, these correlations were not apparent in blood and spleen tissues for peripheral blood viral load (R^2^=0.31 and 0.34, respectively, *P*>0.05) and ALB decline (R^2^=0.04 and 0.17, respectively, *P*>0.05).

### HIV-1 causes pro-inflammatory responses in the liver-immune cell milieu

To evaluate pathomorphological changes produced by HIV-1 infection, we analyzed distribution of human CK18^+^ cells, infiltration with activated HLA-DR^+^ human immune cells, and the presence of HIV-1-infected human cells (p24^+^) in the livers of dual reconstituted mice. We observed diminished CK18^+^ cells in the livers of mice infected with HIV-1 (Fig. 3A, top panel). Both groups of the mice showed significant infiltration by human HLA-DR^+^ cells (Fig. 3A, middle panel), which could be related to the mild form of graft-versus-host disease often observed in animals with high levels of immune reconstitution. The tissue sections were positively stained for p24 (Fig. 3A, bottom panel, Fig. 3B) and the majority of HIV-infected cells (p24^+^ cells) showed lymphocyte morphology, as evidenced by co-staining for CD4 antigen in HIV-infected mice (Fig. 3C). We observed that various degrees of human immune cells infiltration consisted of CD8^+^ and CD4^+^ T cells, macrophages, and very rare B cells positive for IgM/IgG (not shown). However, CD8 cells infiltrating areas occupied by human mismatched hepatocytes were not granzyme B positive. Few granzyme B-positive cells were found in human cell infiltrates containing HIV-1 infected cells (not shown). Dual humanized mice, in comparison to mice transplanted with only Hep, displayed noticeable fibrotic changes, as evident by α-smooth muscle actin (SMA) staining (not shown). The levels of alanine aminotransferase (ALT) were found elevated in all groups of mice exposed to GCV and transplanted with human Hep. Dual reconstituted mice regardless of HIV-1 infection showed higher ALT levels (non-infected median 84 U/l, range 46–473 U/l and infected median 75 U/l, range 25–487 U/l) than hepatocyte-transplanted 70 U/l (range 56–120 U/l, *P*>0.05) and non-humanized mice 26 U/ml (range 13–26 U/l).

**Fig. 4.**
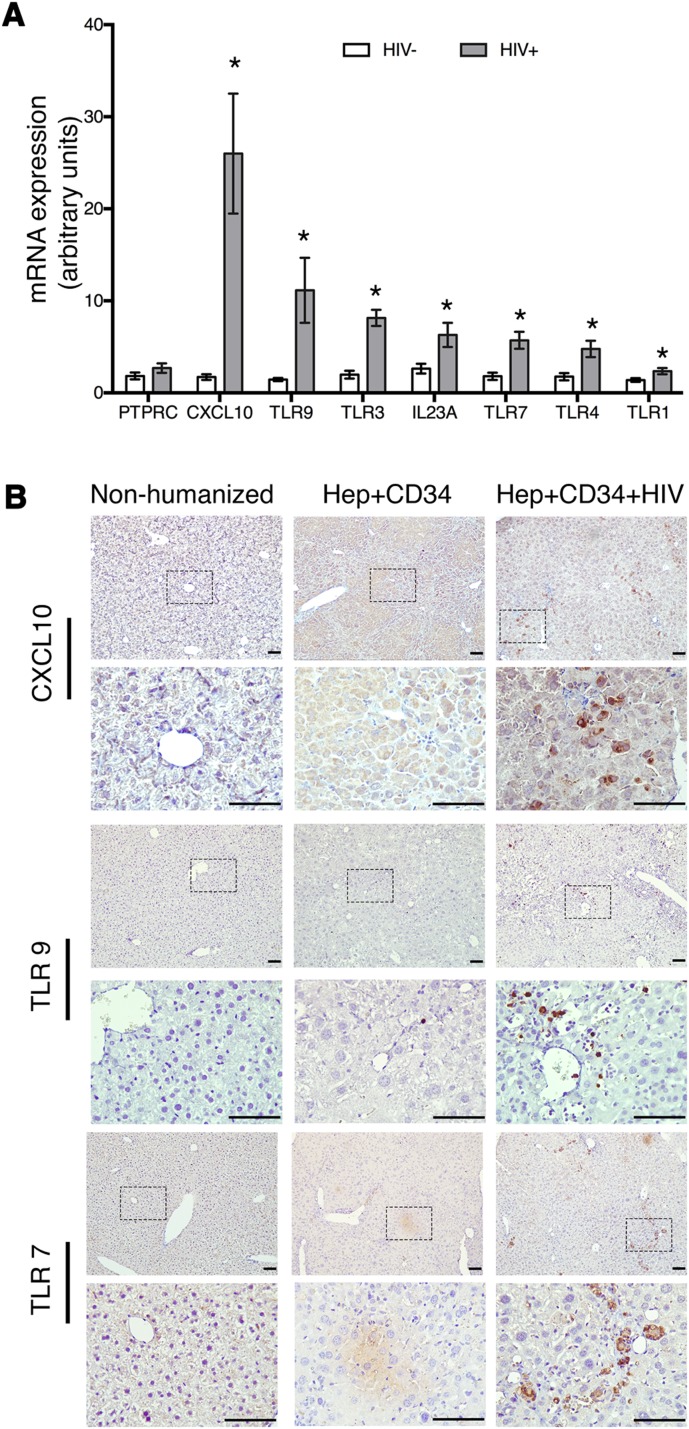
**Upregulation of pro-inflammatory chemokine/cytokines and TLR gene expression in the liver of HIV-1 infected dual reconstituted mice.** (A) Liver mRNA expression of uninfected (HIV–, *n*=8) and HIV-infected (HIV+, *n*=12) mice in 1 μg of total RNA, by comparative CT method. mRNA expression is represented in arbitrary units on the *y*-axis. GAPDH was used as an endogenous control. *P<0.05 by Student's *t*-test, between uninfected and HIV-infected mice. (B) Positive staining of CXCL10, TLR9 and TLR7 antibodies on liver sections of HIV-infected dual humanized mice are shown (right column); staining was not observed in uninfected mice (middle column). The antibodies were human specific and did not show cross reactivity to mouse liver tissue (left column). Liver sections were counterstained with Hematoxylin to demonstrate nuclei. Lower panels corresponding to top panels for each antibody staining are higher magnification images of boxed areas in the top panels. Scale bars: 100 μm.

We observed the decline of human ALB concentration in peripheral blood at 5 weeks post-infection. In euthanized experimental animals we found a significant reduction of the number of human Hep in HIV-1 infected compared to uninfected mice with the exception of two animals (Fig. 1D). To evaluate whether HIV-1 infection induced an inflammatory process in the liver-immune cell milieu, contributing to depletion of Hep and decreased human ALB levels, we investigated the expression profiles of inflammatory chemokines and pattern recognition receptors as a first-line of defense against pathogens. Toll-like receptors (TLRs) trigger innate immunity that results in cellular activation and release of inflammatory factors. We selected transcripts of *TLR9* as a sensor of apoptotic DNA, TLR7 and TLR3 as viral RNA sensors, *TLR4* as LPS sensor, and *TLR1* as a sensor for microbial diacylated and triacylated lipopeptides. Proinflammatory cytokine interleukin *IL-23A* was chosen as a product of pro-inflammatory macrophages responsible for the attraction of neutrophils and the development of pathogenic IL-17-producing T helper cells. Chemokine *CXCL10* was selected as known to be induced by TLR-3 activation in viral infections. We compared the differential transcripts expression between uninfected and HIV-infected mice by RT-PCR; expression was normalized to human-specific *GAPDH*. We detected similar levels of human cells in the livers of both uninfected and HIV-infected mice, as shown by the expression of CD45 encoding *PTPRC* (infected, 2.69±0.51; uninfected, 1.83±0.37) (Fig. 4A). We observed a significant increase in the mRNA expression of alpha chemokine *CXCL10* (26±6.51 versus 1.72±0.30); *TLR9* (11.14±3.38 versus 1.46±0.17), *TLR3* (8.15±0.83 versus 1.98±0.41), *TLR7* (5.71±0.92 versus 1.80±0.37), *TLR4* (4.76±0.88 versus 1.76±0.38) and *TLR1* (2.37±0.33 versus 1.11±0.10); and *IL-23A* (5.34±1.14 versus 2.64±0.53) in the liver of HIV-infected versus uninfected mice. The staining of liver fixed and paraffin-embedded tissue sections with human-specific antibodies to CXCL10, TLR9 and TLR7 confirmed the presence of these proteins in immune cell infiltrates (Fig. 4B). These findings suggest that exposure of human hepatocytes to HIV-1 in combination with the presence of activated and infected human immune cells play a key role in liver damage.

### HIV-1 causes hepatocyte apoptosis in vivo

**Fig. 5.**
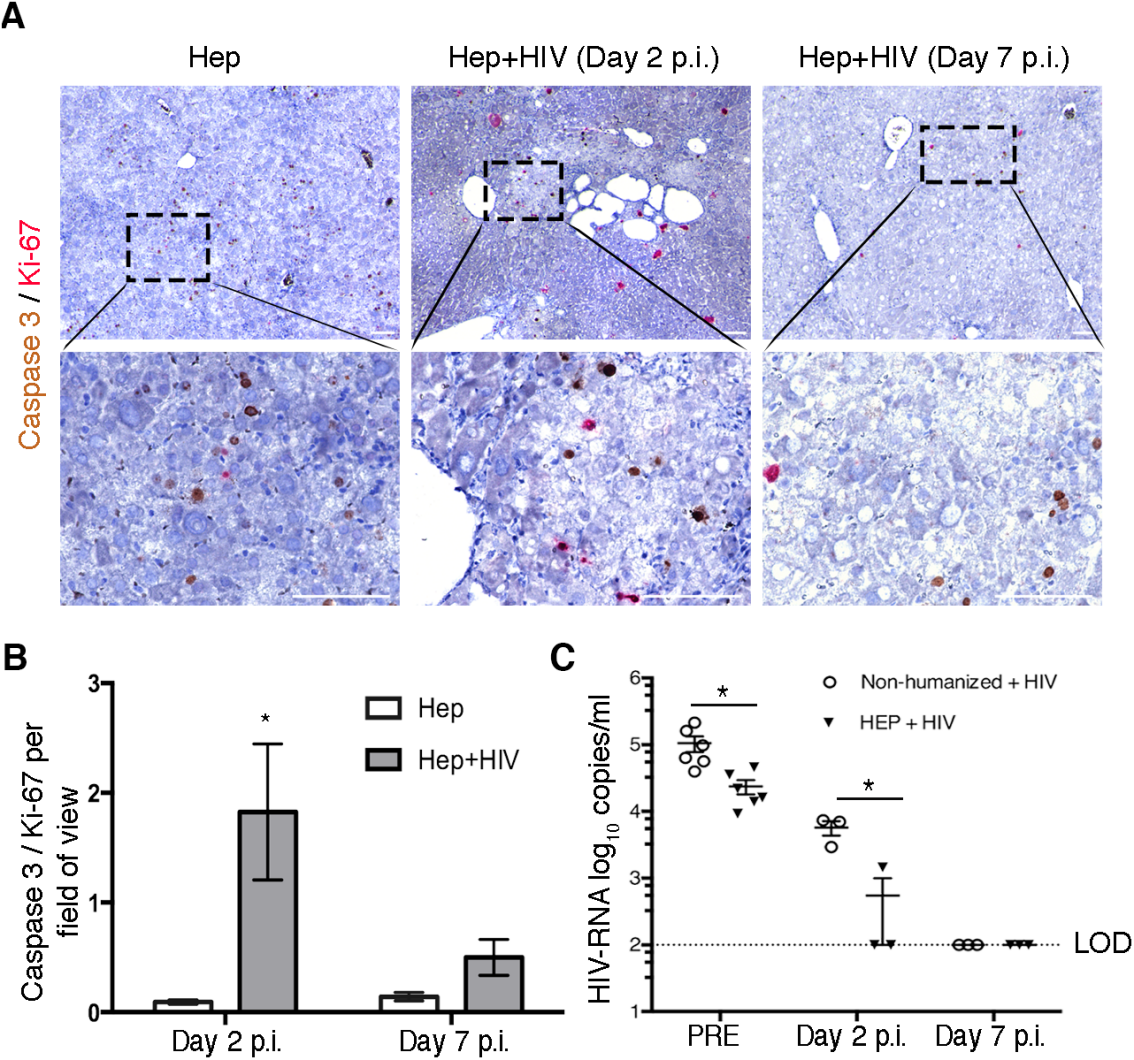
**HIV-1 affects human hepatocyte viability in the absence of human immune system.** Non-humanized and hepatocyte-transplanted TK-NOG mice were infected with high inoculum of HIV-1 3×10^5^ TCID_50_ (*n*=6 per group). HIV-1 infection caused caspase-mediated apoptosis in the liver of HIV-infected hepatocyte-transplanted mice. (A) Liver tissues of uninfected (left column) and HIV-1 infected hepatocyte-transplanted mice at day 2 (middle column, Hep+HIV, *n*=3) and day 7 (right column, *n*=3) were fixed, paraffin embedded, and 5-μm sections were stained for human-specific caspase-3 for apoptosis and Ki-67 for proliferating cells. Caspase-3 and Ki-67 were developed using DAB (brown) and Warp Red (red), respectively, and images were captured on a Nikon Eclipse 55i using a Nuance multispectral imaging system. Bottom row shows the higher magnification images of the boxed areas in the corresponding top row. Scale bars: 100 μm. (B) HIV-RNA copies were detected in the serum of HIV-infected non-humanized (circles, *n*=6) and hepatocyte-transplanted mice (triangles, *n*=6) at day 1 post infection. Mice (*n*=3, each group) were sacrificed on day 2 and day 7. Viral copies in the serum of human Hep transplanted mice declined more efficiently compared to those in non-humanized animals and were below levels of detection at day 2 (*n*=2 out of *n*=3). On day 7, HIV copies were below the limit of detection (LOD) (<log_10_ 2 copies/ml) in both groups of mice. *P<0.05, between non-humanized and hepatocyte-transplanted mice post HIV infection, by Student's *t*-test.

It was shown that HIV-1 and viral proteins can induce human hepatocytes apoptosis *in vitro* (Ganesan et al., 2017a; Munshi et al., 2003; Vlahakis et al., 2003). To evaluate the ability of HIV-1 to induce Hep damage independent from the immune cells we used animals transplanted only with human Hep. Non-humanized TK-NOG mice and hepatocyte-transplanted mice were infected with a high dose of HIV-1 inoculum (3×10^5^ TCID_50_). Caspase-mediated apoptosis of hepatocytes was evaluated by staining for human-specific caspase-3 and normalized to human Ki-67^+^ proliferating cells in the liver of HIV-infected and uninfected hepatocyte-transplanted mice (Fig. 5A). Over 20 fields were randomly selected in a blinded fashion under objective 20× magnification to count all cells positive for caspase-3 and Ki-67, and quantification was represented as ratio of apoptotic cells (caspase-3^+^) to proliferating cells (Ki-67^+^) per field of view. *In vivo* HIV-1 caused apoptosis of human Hep was evident at day 2 post-infection (Fig. 5B); however, apoptosis was reduced and comparable to uninfected mice on day 7. To check HIV-1 kinetics in peripheral blood we collected serum on 1, 2 and 7 days post-infection. To assess viral effect on liver, mice were sacrificed on day 2 and 7 post-infection and viral load in the serum, and HIV*gag* copies in the liver were analyzed. Hep-transplanted mice displayed more rapidly decline of viral copies in the serum at day 2 (*n*=2 out of *n*=3) as compared to non-humanized animals, and viral copies were undetectable by day 7 in both groups of mice (*n*=3 per group; Fig. 5C). We were not able to detect *HIVgag* RNA in the in the liver of both groups of mice at day 2 and day 7. Furthermore, human ALB and ALT levels in these experimental animals were not affected by HIV infection (not shown).

## DISCUSSION

Rodent models that accurately recapitulate liver involvement in HIV-1 immunopathogenesis are essential for research and therapeutic development. Mice transplanted with human CD34^+^ HSPCs to study HIV-1 infection are valuable tools to address questions about HIV-1 pathobiology and treatment. For example, in mice transplanted with CD34^+^ HSPCs, infiltration of immune cells contributed to fibrosis of the liver (Nunoya et al., 2014). Moreover, chronic infection (more than 16 weeks) led to decreased mouse ALB levels, and successful treatment with long-acting antiretroviral drugs restored synthetic mouse liver function (Dash et al., 2012). These data complement *in vivo* reports that human immune cells contribute to HIV-1-specific liver pathogenesis, but the effect on human, not mouse Hep was lacking. The species specificity of immune and functional properties of mouse and human Hep could underline the differences in Hep responses to HIV-1 and immune mechanisms. To understand this effect and to elucidate the pathogenesis and immunological consequences of HIV-induced parenchymal damage, we engrafted TK-NOG mice with both human Hep and CD34^+^ HSPCs (Hasegawa et al., 2011; Ito et al., 2002; Kosaka et al., 2013).

Dual humanized mice represent a reasonable tool for HIV studies. We clearly realize that this model also has some limitations, such as transplantation of Hep in combination with a mismatched immune system. This could not be avoided, given that hepatocytes and immune cells from the same donor are not usually available. Only a few reports of co-transplantation of matched human Hep and HSPC addressed various aspects of hepatotropic human-specific infections, such as hepatitis C and B (Bility et al., 2013; Billerbeck et al., 2016; Washburn et al., 2011). In both studies the levels of human ALB (reflection of the number of engrafted Hep) were low (in µg/ml) and fates of Hep was not evaluated, while the infiltration of human immune cells and fibrotic changes were noted. Human-specific inflammatory pathways contributing to hepatocyte death and the interaction of activated immune cells with hepatocytes could thus be addressed in the presence of all human immune cells (myeloid, NK, T and B cells) in the mouse model with non-autologous human liver and immune cells.

In the present study, single-donor Hep was transplanted with CD34^+^ cells from different single donors. The main focus of the study was to investigate the immunopathological responses that occur in dual reconstituted humanized mice, monitoring the survival and function of human Hep based on levels of secreted human ALB. In our studies, the mismatched immune cells did not contribute to elimination of non-autologous hepatocytes (similar for all animals) and HIV-1 infection unexpectedly rushed the depletion of engrafted cells. Survival and expansion of human Hep in such a model is a complex process in which the amount of mouse hepatocytes depleted by GCV is a critical component. Unfortunately, almost all TK-NOG mice that received GCV showed regenerative nodule derived from mouse hepatocytes, slightly elevated ALT and some mice showed cystic structures at 6 months of age. Surprisingly, the transplantation of Hep with mismatched CD34^+^ cells did not preclude expansion of Hep and this could be the cause of higher ALB levels in mice co-transplanted with CD34^+^ cells than that found in hepatocyte-transplanted mice (Fig. 1B). In animal models, immune cells have been shown to facilitate the expansion and differentiation to hepatocytes (Almeida-Porada et al., 2004; Hu et al., 2016; Streetz et al., 2008). As demonstrated earlier, despite the presence of human CD8^+^ cells, transplantation of mismatched human cells displayed no rejection for allogeneic Hep (Gutti et al., 2014; Strick-Marchand et al., 2015). These observations corroborated experimental allogeneic co-transplantation of bone marrow and hepatocytes in mouse models, where CD4^+^ cells played an essential role in supporting hepatocytes expansion (Streetz et al., 2008).

In our experimental animals transplanted with mismatched CD34^+^ cells, Hep rejection was not observed, but we noted that human ALB levels decline at 5 weeks post-HIV-1 infection. Decline in ALB was corroborated to significant reduction of human Hep population (Fig. 1C). The degree of ALB reduction in our mouse model correlated with increased HIV-1 viral load in peripheral blood (Fig. 1D). Numerous clinical observations of mono-infected patients have shown that the serum ALB level is a useful marker of HIV-1-associated liver disease progression (Dusingize et al., 2015; Graham et al., 2007; Mehta et al., 2006). An observed drop in human ALB levels could be attributed to multiple mechanisms, such as the reduction of synthetic activity of Hep, inflammation and immune activation, as well as Hep death (Giannini et al., 2005; Hu et al., 1992; Mehta et al., 2006). We believe that the presence of immune cell infiltrates in the liver, pro-inflammatory cytokines, and pro-apoptotic ligands secreted by HIV-1 infection-activated immune cells contributed significantly to hepatocyte death (Crane et al., 2012). Consistent with previous studies, HIV-infected mice displayed significant reduction in CD4^+^ cells in blood, spleen and liver tissues (Lane, 2010). Here, we observed a strong correlation of decline in CD4^+^/CD8^+^ ratios with decreased levels of human ALB (Fig. 2E).

To test the possibility of liver-immune cell activation by HIV-1, we compared expression of pro-apoptotic and pro-inflammatory cytokines and chemokines, and TLRs in the liver of infected versus uninfected mice dual transplanted mice using RT-PCR and immunohistochemistry. There was evident upregulation of transcripts of *CXCL10*, *IL23A*, *TLR9*, *TLR3*, *TLR7*, *TLR4* and *TLR1* in HIV-1-infected animals compared to uninfected mice. Similar observations were reported in a clinical setting when liver specimens from uninfected, HCV-infected and HIV/HCV co-infected patients were analyzed (Hu et al., 2014) and reviewed (Nakamoto and Kanai, 2014). *TLR7* and *TLR9* have been shown to induce immune activation resembling HIV-mediated pathology in humanized mice (Baenziger et al., 2009), and *TLR9* activation by DNA products is hepatotoxic (Heikenwalder et al., 2004). Activation of immune cells in combination with HIV-1-induced Hep death may be partially triggered by liver macrophage engulfment of hepatocyte-derived apoptotic bodies by liver macrophages, which induce liver inflammation by secretion of pro-inflammatory cytokines. A similar mechanism was previously established in HCV-infected hepatocytes and their interactions with macrophages (Ganesan et al., 2016). In HIV-infected patients, LPS stimulated TLR4 was shown to deplete CD4^+^ T cells by T-cell activation (Brenchley et al., 2006). Here, we demonstrated an increase in transcript of proinflammatory interleukin *IL-23A*, which is associated with HIV-1-induced disease progression in patients (Louis et al., 2010). We also observed increased positive staining of alpha chemokine *CXCL10* (Fig. 4B), a well-known marker of chronic liver inflammation in HIV/HCV co-infection that shows a *TLR4*-mediated pro-apoptotic effect on hepatocytes (Sahin et al., 2013). Activated immune cells induce Fas-L and upregulate Fas, TNF and TRAIL to induce apoptosis in the cells (Ju et al., 1995; Nagata, 1997); and infection of HIV-1 can further exaggerate hepatocytes death by triggering TLR-mediated innate immune response and by elevated proinflammatory cytokines in the liver-immune cell milieu (Sahin et al., 2013). Unfortunately, by 5 weeks post HIV-1 infection, we did not have human hepatocytes in the dual humanized mice livers to observe the expression of possible receptors and/or ligands on human hepatocytes.

Irrespective of HIV-infection, the model also has some limitations such as inefficiency of human adaptive immune responses: low levels of cytotoxic T cell activity and in immunoglobulin production and impaired immunoglobulin class switching. The utility for studying liver fibrosis of this model is also limited due to noticeable αSMA expression in immune system reconstituted mice. As was previously shown, HIV-1 infection alone did not induce significant fibrotic changes in Rag2^−/−^ IL-2Rγ^−/−^ or NOD-scid IL-2Rγ^−/−^ reconstituted with immune system only; however, the depletion of regulatory T cells and significant influx of human immune cells and macrophages trigger inflammation and fibrosis (Nunoya et al., 2014). Animals in our studies had variable immune cell infiltration, but HIV-1 infection did not statistically significantly change the number of infiltrating cells that was confirmed by RT-PCR expression of human CD45 (Fig. 4A).

Further, due to loss of major histocompatibility complex (MHC)-matched interaction between hepatocytes and immune cells, adaptive cellular immunity studies could be limited in this mouse model for hepatitis B/C viruses that productively infect human hepatocytes. However, the model is not disadvantageous for HIV-1, given that infection of hepatocytes is not productive and that viral nucleic acids can be detected by Hep and immune cells TLRs. Finally, we explored the ability of HIV-1 to trigger apoptotic death directly in the absence of human immune cells. We infected hepatocyte-transplanted animals with high HIV-1 inoculum and evaluated apoptosis by staining for caspase-3 and Ki-67. We observed increased staining for caspase-3 at day 2, and by day 7, apoptosis in HIV-infected mice was comparable to that in uninfected mice (Fig. 5). Based on the data acquired by liver histology of HIV-1-infected dual humanized TK-NOG mice, apoptosis could be a cause of hepatocyte death and the consistent presence of infection and immune activation will eventually impair the ability of human Hep to proliferate.

We have explored the immunopathogenesis of HIV-1-induced liver disease in HIV-1-infected dual liver and immune system reconstituted mice. The model recapitulates multiple components of liver damage by HIV-1 infection as in humans, including: (1) HIV-1-induced depletion in liver CD4^+^ cells; (2) decreased ALB levels; (3) liver immune activation; and (4) human hepatocyte death. Thus, the resemblance of HIV-1 infection to clinical findings in the dual human liver and immune system TK-NOG mouse model multiplies experimental possibilities for the study of HIV-1 infection. Indeed, interaction studies of the human immune system and liver pathology could be essential for co-infection studies of hepatitis viruses and the evaluation of antiretroviral drugs.

## MATERIALS AND METHODS

### Immune system and liver humanization of TK-NOG mice

TK-NOG (NOD.Cg-Prkdc^scid^ Il2rg^tm1Sug^ Tg(Alb-TK)7-2/ShiJic) mice expressing a liver-specific herpes simplex virus type 1 *TK* transgene under the control of an ALB promoter were provided by the Central Institute for Experimental Animals (CIEA, Japan; Drs Mamoru Ito and Hiroshi Suemizu) (Ganesan et al., 2017b). Mice were bred and housed in the pathogen-free animal facility at the University of Nebraska Medical Center (UNMC). Human umbilical cord blood was collected after delivery (Department of Obstetrics and Gynecology, UNMC) and processed as previously described (Gutti et al., 2014). Human CD34^+^ HSPCs were isolated from umbilical cord blood or fetal liver using the human CD34 microbead kit (Catalog 130-046-702, Miltenyi Biotec, Auburn, AL, USA), as per the manufacturer’s instructions, and the purity of isolated cells was evaluated by flow cytometry (∼90%). CD34^+^ HSPCs were frozen for future transplantation. Plateable, induction-qualified cryopreserved human Hep from a single donor was purchased from Triangle Research Labs (Catalog HUCP1, Lot HUM4051, TRL, Research Triangle Park, NC, USA). TK-NOG mice were genotyped as published (Hasegawa et al., 2011). TK-NOG males (6-8 weeks) were injected with GCV to deplete transgenic liver parenchymal cells, as previously described (Kosaka et al., 2013) and animals with ALT levels >200 U/ml were selected for human hepatocyte transplantation. For double transplantation of CD34^+^ HSPCs and Hep, mice were preconditioned with an intraperitoneal injection of treosulfan (kind gift from Dr Joachim Baumgart, Medac GmbH, Hamburg, Germany) for 3 days at a dose of 1.5 g/kg/day. Animals listed in Table S1 were transplanted with the mixture of MHC mismatched single-donor human 10^6^ Hep and 10^5^ CD34^+^ HSPCs derived from eight single donors per mouse via intrasplenic infusion (Dagur et al., 2017; Gutti et al., 2014). Mice with Hep and not injected with hCD34^+^ HSPCs were considered as control to dual reconstituted mice (Table S2). The experimental design for dual reconstitution of mice is illustrated in Fig. 1A.

### HIV-1 infection

HIV-1_ADA_ was propagated on human monocyte-derived macrophages as previously described (Gendelman et al., 1988). An indicator cell line TZM-bl (JC53-bl, clone 13) was obtained from the NIH AIDS Reagent Program (Catalog 8129) and used for viral stock titration according to standard protocol, as previously described (Montefiori, 2009). TK-NOG males with human CD45 levels >15% and presence of human ALB in peripheral blood were intraperitoneally injected with HIV-1_ADA_, a CCR5-utilizing virus at a dosage of 10^4^ TCID_50_/mouse at 5 months post-transplantation. Dual reconstituted mice injected with phosphate buffered saline (PBS) only were considered uninfected. The level of viral replication was analyzed in serum by using an automated COBAS Amplicor System v1.5 (Roche Molecular Diagnostics), as previously described (Dash et al., 2012). Non-humanized and hepatocyte-transplanted mice were infected with a high dose of HIV-1 inoculum (3×10^5^ TCID_50_).

### Immunophenotyping by flow cytometry

Human immune reconstitution was evaluated by flow cytometry (FACS) in CD34^+^ HSPC recipient TK-NOG mice at 1, 3 and 4 months post-transplantation and 5 weeks post-infection. Blood samples were collected from the submandibular vein by lancets (MEDIpoint, Mineola, NY, USA) in EDTA tubes. After euthanizing mice, liver and spleen tissues were isolated and single cell suspensions of samples were prepared by mechanical crushing and passing through a 40 μm nylon mesh strainer (BD Biosciences). Exclusion of hepatocytes was performed by centrifugation at 300 rpm for 3 min. Non-parenchymal cells in supernatant were centrifuged at 1500 rpm for 10 min at 4°C. Pellet was suspended in FACS buffer (PBS supplemented with 2% FBS). Single cell suspensions from blood, spleen, and liver were suspended in FACS buffer and were stained for mouse CD45 and human antibodies (CD45, CD3, CD4, CD8, CD19) for 30 min at 4°C as six-color combinations (Table S2), followed by removal of red blood cells with FACS Lysing solution (BD Biosciences). Acquisition of stained cells was performed on BD LSR II (BD Biosciences) using acquisition software FACS Diva v6 (BD Biosciences), and data was analyzed using FLOWJO analysis software v10.2 (https://www.flowjo.com/solutions/flowjo/downloads).

### Human albumin quantitation by ELISA

The efficiency of Hep engraftment was evaluated every month via quantifying human ALB within mouse serum, using human Albumin ELISA Quantitation set (Catalog E80-129, Bethyl Laboratories, Montgomery, TX, USA) as per the manufacturer's instructions. Absorbance was read at 450 nm on a SpectraMax M3 (Molecular Devices).

### Histopathology evaluation by immunohistochemistry

Tissues were fixed with 4% paraformaldehyde (PFA) overnight and embedded in paraffin. Five-micron thin sections were cut from the paraffin blocks, mounted on glass slides, and subjected to immunohistochemical staining with mouse monoclonal antibodies for human leukocyte antigen-DP, DQ, DR (HLA-DR), HIV-1 p24, cytokeratin-18 (CK18) and TLR7; rabbit monoclonal CD4 (Abcam); rabbit polyclonal and mouse monoclonal caspase-3+Ki-67 (Biocare Medical, Pacheco, CA, USA); rat monoclonal TLR9; and goat polyclonal CXCL10. A list of antibodies and working dilutions is provided in Table S3. Fluorescent secondary antibodies or polymer-based HRP conjugated anti-mouse or anti-rabbit Dako EnVision systems (Dako, Carpinteria, CA, USA) or anti-goat (R&D Systems) and MACH2 double stain 2 (Biocare Medical) were used as secondary detection reagents and were developed with DAB, and Permanent Red (Dako) or Warp Red (Biocare Medical). All paraffin-embedded sections were counterstained with Mayer's Hematoxylin (Leica, Allendale, NJ, USA). Images were obtained on Nikon Eclipse 55i (Nikon Instruments, Melville, NY, USA) using a Nuance multispectral imaging system FX (Perkin Elmer, Waltham, MA, USA) and evaluated by two investigators blinded to the identity of the specimens. Caspase-mediated apoptosis of hepatocytes was evaluated by staining for human-specific caspase-3 and normalized to human Ki-67^+^ proliferating cells in the liver. Over 20 fields were randomly selected in a blinded fashion under objective 20× magnification on a Nikon Eclipse E400 brightfield microscope (Nikon Instruments) to count all cells positive for caspase-3 and Ki-67, and quantification was represented as ratio of apoptotic cells (caspase-3^+^) to proliferating cells (Ki-67^+^) per field of view.

### Semi-nested real-time PCR

Liver tissues from infected mice (Tables S1 and S2) were homogenized to isolate total RNA using an RNeasy Plus Universal Kit (Qiagen) as per the manufacturer's instructions. An equal amount of 1 µg RNA was used to reverse-transcribe into cDNA, using a cDNA synthesis kit (Thermo Fisher Scientific), following the manufacturer's instruction. Semi-nested real-time PCR was performed as described earlier, and included two rounds of PCR amplification of the HIV*gag* region (Araínga et al., 2016; Pasternak et al., 2008). Briefly, the first PCR was performed in a S1000™ Thermal Cycler (Bio-Rad) in a 25 µl mix containing 8 µl of cDNA sample, 1 µl of 10 µm *gag* sense primer (5′-TCAGCCCAGAAGTAATACCCATGT-3′), 1 µl of 10 µm *gag* antisense SK431 primer (5′-TGCTATGTCAGTTCCCCTTGGTTCTCT-3′), 12.5 µl Amplitaq Gold 360 master mix, and 2.5 µl enhancer purchased from Applied Biosystems. Cycling parameters were as follows: 94°C for 3 min, followed by 15 cycles of 94°C for 30 s, and 72°C for 1 min. The first PCR product was subsequently used as a template and amplified on a StepOne Plus™ Real-Time PCR (Applied Biosystems) with HIV1*gag* primer-probe set (sense: 5′-TCAGCCCAGAAGTAATACCCATGT-3′; antisense 5′-CACTGTGTTTAGCATGGTGTTT-3′; TAMRA-labeled probe FAM-ATTATCAGAAGGAGCCACCCCACAAGA-TAMRA) as previously described (Araínga et al., 2016). Real-time PCR settings were as follows: 50°C for 2 min, then 95°C for 10 min, followed by 40 cycles of 95°C for 15 s and 60°C for 1 min. ACH2 cells obtained through the NIH AIDS Reagent program (Catalog 349) contained one integrated copy of HIV per cell, and were used as standards as described previously (Araínga et al., 2016). ACH2 cell DNA was used to construct a dilution series ranging from 10×10^4^ to 10 DNA copies per sample. Human *GAPDH* (Catalog 4326317E, Applied Biosystems) was used as an endogenous control. Copy numbers for transcripts of HIV*gag* were calculated by relative standard curve method and expressed as HIV*gag* copies in 1 µg of RNA. Uninfected liver tissue samples obtained from humanized and non-humanized animals were used as controls and did not show amplification.

### Real-time PCR

To test the mRNA expression of pro-inflammatory cytokines, RNA and cDNA were prepared as described above in the semi-nested PCR, and amplification was performed on a StepOne Plus™ Real-Time PCR (Applied Biosystems). Human-specific primer-probe sets (*PTPRC*, Hs04189704_m1; *CXCL10*, Hs01124252_g1; *TLR9*, Hs00370913_s1; *TLR3*, Hs00152933_m1; *IL23A*, Hs00413259_m1; *TLR7*, Hs01933259_s1; *TLR4*, Hs00370853_m1; *TLR1*, Hs00413978_m1) and *GAPDH* (Hs99999905_m1) were purchased from Applied Biosystems. Expression values were presented in arbitrary units calculated using the comparative threshold cycle method after normalization with an endogenous human *GAPDH* transcript expression of total RNA (Teotia et al., 2017). The primers were human-specific as observed by BLAST analysis. No amplification was observed on non-humanized mice livers, confirmed by real-time PCR (data not shown).

### Statistics

Data were analyzed and plotted using GraphPad Prism and expressed as mean±s.e.m. or median and range. Statistical significance was calculated by one-way or two-way ANOVA or Student's *t*-test and indicated in the respective figure legends. Correlation between variables was evaluated by Pearson's correlation. *P*<0.05 was considered significant.

### Study approval

Animal studies were carried out in accordance with the guidelines for humane care of laboratory animals. The UNMC Animal Care and Use Committee approved all experimental protocols. Human cord blood samples were obtained after receiving informed consent from parents and then approved by the Institutional Review Board (IRB) of the University of Nebraska Medical Center. Human fetal liver samples were obtained from the University of Washington Medical Center Laboratory of Developmental Biology (R24HD000836-51).

## Supplementary Material

Supplementary information

## Supplementary Material

First Person interview
