# Supplementary material for: Human hepatocyte depletion in the presence of HIV-1 infection in dual reconstituted humanized mice
Source: Biol Open. 2018 Jan 22;7(2):bio029785. doi: 10.1242/bio.029785 (PMC5861361; doi:10.1242/bio.029785)
Supplement: Supplementary information [file biolopen-7-029785-s1.pdf]

Supplemental Material

Table S1. Characteristics of dual humanized mice.

Experimental groups	Mouse ID	$CD34^+$ cells donor	Human CD45, %	Albumin, µg/ml	HIV-RNA copies/ml or HIV-1 $P24^+$ by IHC
HIV-infected (Hep+CD34+HIV) mice	1265	A	31.6	49.1	3990000
	1263	A	45.3	ND*	3130000
	3217	B	46.2	593	2270
	3218	B	47.3	391	HIV-1 $P24^+$ cells†
	3123‡	C	49.6	169	1221000
	1283	A	55.5	13.1	3160000
	3191	H	74.6	88	2090000
	3224	G	74.7	0	2629000
	3209	G	84.4	ND*	HIV-1 $P24^+$ cells†
	3274	F	70.9	ND*	HIV-1 $P24^+$ cells†
	2188	D	ND§	180	75500
	1262	A	ND§	118.8	635000
	3189	D	ND§	287	111000
Uninfected (Hep+CD34) mice	1297	E	91.7	359.8	-
	2174	F	92	148.7	-
	2141	F	16	119.8	-
	493	H	28	1083.6	-
	3128‡	C	62.6	779.5	-
	3129‡	C	ND§	664	-
	3376	G	66.7	336	-
	2165	F	ND§	117.6	-
	490	H	ND§	1653	-
	2090	H	ND§	2663	-
	1282	A	23.8	330	-
Uninfected (Hep) mice	3006	–¶	–¶	713.4	-
	2166	–¶	–¶	1215.7	-
	3262	–¶	–¶	161.83	-
	1291	–¶	–¶	956	-
	3024	–¶	–¶	403	-
	3033	–¶	–¶	708	-
	3044	–¶	–¶	312	-

*, ND for no data collection of albumin by ELISA; †, HIV-1 infection was detected by strong positive staining of infected cells in spleen and liver tissues; ‡, Animals reconstituted with the donor 'C' $CD34^+$ cells and two uninfected mice experienced albumin reduction regardless of HIV-1 infection so albumin drop in HIV-infected mouse could be other than HIV-1 infection and were excluded from albumin and correlation analysis; §, ND for

Biology Open • Supplementary information

no data collection at the end for hCD45$^+$ cells in blood by flow cytometry, humanization was confirmed by immunohistology and FACS analysis performed previously on mice prior to sacrifice; -, Mice were not exposed to HIV-1 so HIV-RNA copies and HIV P24$^+$ cells were not tested; _¶, Mice were not injected with CD34$^+$ cells so not tested for the presence of human CD45$^+$ cells in the blood.

TK-NOG males were transplanted with both hepatocytes (Hep) and CD34$^+$ hematopoietic stem/progenitor cells (CD34$^+$HSPC) and considered as dual humanized mice (Hep+CD34). All mice were transplanted with commercially available single donor Hep, purchased from Triangle Research Labs, USA. CD34$^+$HSPC were isolated from multiple donors by positive selection. Hep+CD34 mice were infected with HIV-1_{ADA} (Hep+CD34+HIV; *n*=13), and HIV-1 infection was confirmed by the presence of HIV-RNA copies in serum and HIV P24$^+$ cells in the liver and spleen tissues. Uninfected Hep+CD34 mice (*n*=11) were control to HIV-infected dual humanized mice. Five-weeks post-infection, dual humanized mice were checked for human immune system reconstitution by presence of CD45$^+$ cells in peripheral blood and liver engraftment by serum albumin levels. Hepatocytes-transplanted mice were Hep mice (*n*=7) and served control to dual humanized mice. Hep mice were also sacrificed at the same time and checked for serum albumin levels for liver engraftment.

Biology Open • Supplementary information

Table S2: Albumin levels and viral load of uninfected hepatocyte-transplanted (Hep) mice, and HIV-infected Hep mice and non-transplanted mice.

Experimental groups	Mouse ID	Albumin, µg/ml	HIV-RNA copies/ml (viral load)
**Uninfected (Hep) mice**	3777*	123.6	-
	3779*	122.5	-
	3517*	83.5	-
	4011†	141.7	-
	4015†	114.1	-
**HIV-infected (Hep) mice**	3755*	858.1	1448
	3757*	1038.7	Neg
	4012*	773.5	Neg
	3760†	1058.2	Neg
	3761†	1539.1	Neg
	3778†	774.8	Neg
**HIV-infected non-humanized mice**	247*	57.9	6897
	248*	71.9	7344
	249*	80.8	2938
	250†	Neg	Neg
	251†	Neg	Neg
	252†	Neg	Neg

*, Mice were sacrificed on day 2 post HIV-infection; -, Mice were never exposed to HIV-1 and HIV-RNA copies were not tested; †, Mice were sacrificed on day 7 post HIV-1 infection; Neg, below detection limit.

TK-NOG males were transplanted with hepatocytes (Hep) and infected with HIV-1. Uninfected hepatocyte-transplanted mice and HIV-infected non-transplanted (non-humanized) mice served as controls. All mice from hepatocyte-transplanted mice group were transplanted with commercially available single donor Hep, purchased from Triangle Research Labs, USA. HIV-1 infection was confirmed on day 2 and day 7 post HIV-infection by the presence of HIV-RNA copies in serum. Post-infection on day 2 and day 7, mice were also checked for serum albumin levels and presence of HIV-RNA copies in serum. Albumin was detectable on day 2 in serum of non-humanized mice due to traces of human albumin in HIV-1 viral stock medium.

Table S3: List of primary antibodies used for flow cytometry, immunocytochemistry and immunohistochemistry.

Antibodies and reagents	Cross-reactivity	Host	Dilution	Company	Catalog number
CD45-FITC	Mouse	Rat	5µl/test	BD Biosciences	553080
CD45-PerCP-Cy5.5	Human	Mouse	10µl/test	BD Biosciences	564105
CD3-AF700	Human	Mouse	5µl/test	BD Biosciences	557943
CD19-BV605	Human	Mouse	5µl/test	BD Biosciences	562653
CD4-APC	Human	Mouse	5µl/test	BD Biosciences	555349
CD8-BV421	Human	Mouse	5µl/test	BD Biosciences	562428
HLA-DP, DQ, DR	Human	Mouse	1:200	DAKO	M0775
HIV-1 p24	Human	Mouse	1:10	Dako	M0857
Caspase-3+Ki-67	Human	Rabbit / Mouse	Prediluted	Biocare Medical	PPM240DSAA
CXCL10	Human	Goat	1:25	R&D Systems	AF-266-SP
TLR9	Human	Rat	1:25	LifeSpan Biosciences	LS-B7876
TLR7	Human	Mouse	1:25	LifeSpan Biosciences	LS-C358104
CD4	Human	Rabbit	1:500	Abcam	ab133616

Biology Open • Supplementary information

Supplemental materials

Fig. S1

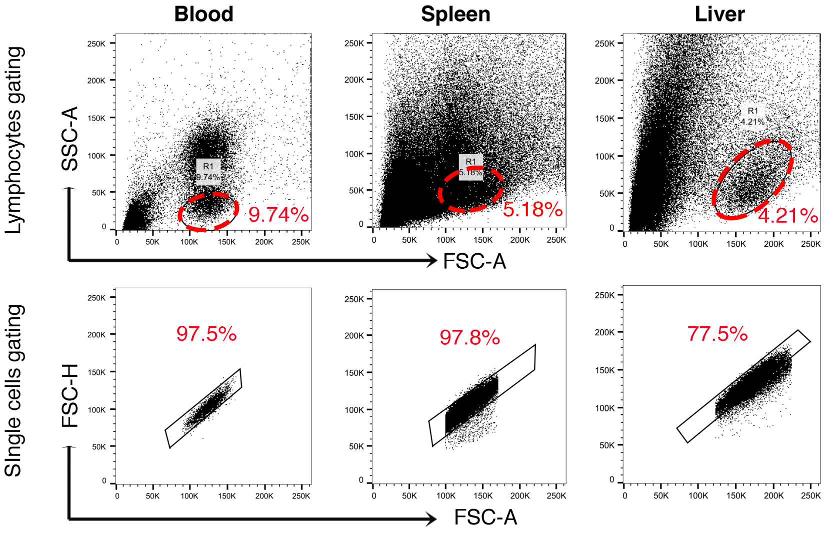

Fig. S1. Representative gating strategy for immunophenotyping of blood, spleen and liver. Regions were selected to gate lymphocytes (ellipse) on the basis of scatter pattern on forward scatter (FSC) versus side scatter (SSC) plots. Single cells were gated on lymphocytes to exclude doublets on FSC-A versus FSC-H dot plots. This gating strategy was used to characterize various immune cells in Fig. 3.
