# Supplementary material for: Human hepatocyte depletion in the presence of HIV-1 infection in dual reconstituted humanized mice
Source: Biol Open. 2018 Jan 22;7(2):bio029785. doi: 10.1242/bio.029785 (PMC5861361; doi:10.1242/bio.029785)
Supplement: First Person interview [file biolopen-7-029785-s2.pdf]

© 2018. Published by The Company of Biologists Ltd | Biology Open (2018): doi:10.1242/bio.029785: Supplementary information

FIRST PERSON

First person – Raghubendra Dagur

First Person is a series of interviews with the first authors of a selection of papers published in Biology Open, helping early-career researchers promote themselves alongside their papers. Raghubendra Dagur is first author on 'Human hepatocyte depletion in the presence of HIV-1 infection in dual reconstituted humanized mice', published in BiO. Raghubendra is a Postdoctoral Research Associate in the lab of Larisa Poluektova in the Department of Pharmacology and Experimental Neuroscience at University of Nebraska Medical Center, USA, investigating humanized mice for translational research.

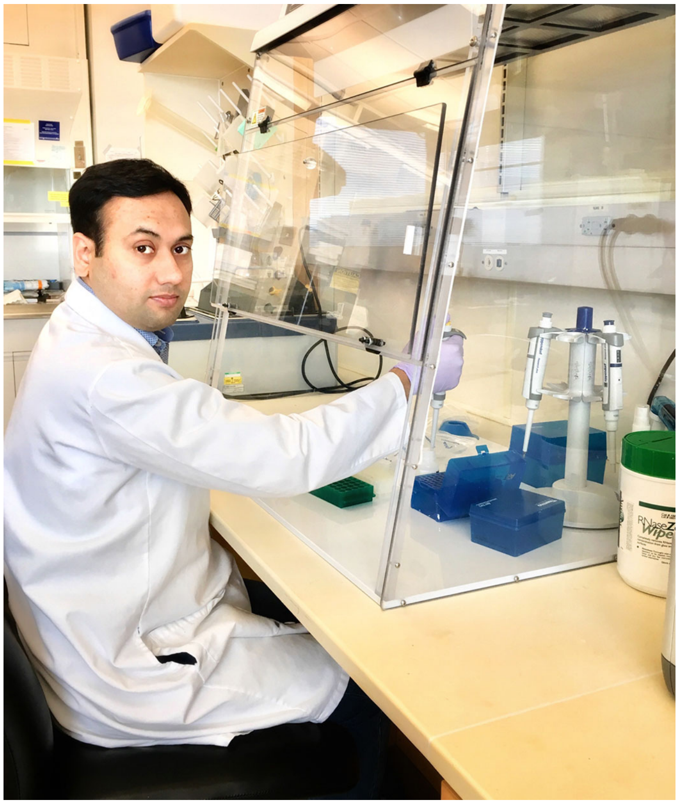

Raghubendra Dagur

What is your scientific background and the general focus of your lab?

My academic training and research experience during graduation have provided an excellent background in multiple biological disciplines, including cell biology, molecular biology and radiation biology. My particular interest in preclinical research led me to move to Drs Poluektova and Gorantla's research group at University of Nebraska Medical Center as a postdoctoral research associate. Our laboratory is particularly interested in developing small 'humanized' animal models for HIV-1, hepatitis B and C pathogenesis, therapeutics and vaccines, stem cell transplantation and other translational research.

How would you explain the main findings of your paper to non-scientific family and friends?

We have utilized a mouse model that has both a functional human immune system and liver to study liver damage as observed in HIV-1-infected patients. The mouse model could be used in co-infection studies of hepatitis and HIV-1 viruses, and in the evaluation of antiretroviral drugs.

> "One of the major problems for early-career scientists is lack of funding opportunities and huge competition for limited funds."

What are the potential implications of these results for your field of research?

Our study showed multiple mechanisms of liver damage by HIV-1 infection as in humans, including: 1) HIV-1-induced depletion in liver CD4+ cells; 2) decreased human albumin levels; 3) liver-immune activation; and 4) human hepatocyte death. Thus, the resemblance of HIV-1 infection to clinical findings in the dual human liver and immune system in the TK-NOG mouse model multiplies experimental possibilities for the study of HIV-1 infection. Mice reconstituted with a dual human immune system and liver cells could be beneficial for interaction studies of the human immune system and liver pathology, which could be essential for co-infection studies of hepatitis viruses (hepatitis B and C) and the evaluation of antiretroviral drugs.

What has surprised you the most while conducting your research?

The development of a humanized mouse model with a dual human immune system and hepatocytes is challenging due to non-availability of a matched donor, eventual graft versus host disease, and the overall fragility of humanized mice. Surprisingly, the TK-NOG mouse model did not show rejection of transplanted mismatched hepatocytes by the developed human immune system and quite remarkably supported liver engraftment, which led us to show the effects of HIV-1 on the liver. Preclinical testing on humanized mice is required before human clinical trials can be conducted due to ethical concerns.

What, in your opinion, are some of the greatest achievements in your field and how has this influenced your research?

Although several new animal models have been developed for better understanding of human physiology, the best strains of mice for dual reconstitution with available tissue sources are still limited. Mouse models with an NOG background (NOD/Shi-scid/IL-2Rgcnull) are superior to other existing models for engraftment of human immune system and liver. Based on that,

Raghubendra Dagur's contact details: Department of Pharmacology and Experimental Neuroscience, University of Nebraska Medical Center, Omaha, USA.
E-mail: raghu.dagur@unmc.edu

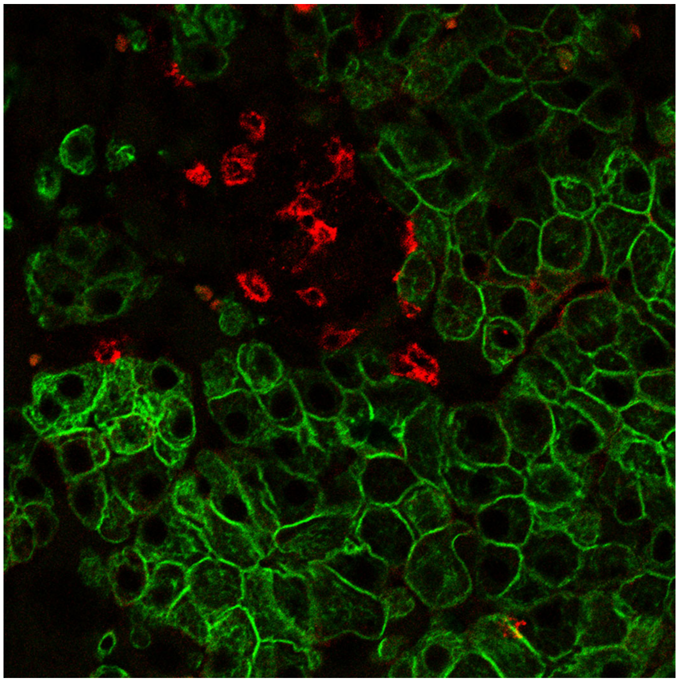

Immunofluorescence staining of a dual humanized mouse liver section for human immune cells (CD8, red) and hepatocytes (CK18, green).

we utilized the TK-NOG mouse model that expressed thymidine kinase under control of a liver-restricted albumin promoter to monitor liver reconstitution. The TK-NOG mouse model showed robust engraftment of both human immune system and liver, letting us explore the HIV-1-associated liver immunopathogenesis.

What changes do you think could improve the professional lives of early-career scientists?

One of the major problems for early-career scientists is lack of funding opportunities and huge competition for limited funds, especially for those who are international students. Perpetual support from mentors is crucial to improve the professional development of early-career scientists.

What's next for you?

I am currently looking for an early-career faculty position, where I can use my skills that I have developed during the postdoctoral training, and continue research on disease modeling and humanized mouse model development for translational studies.

Reference

Dagur, R. S., Wang, W., Cheng, Y., Makarov, E., Ganesan, M., Suemizu, H., Gebhart, C. L., Gorantla, S., Osna, N. and Poluektova, L. Y. (2018). Human hepatocyte depletion in the presence of HIV-1 infection in dual reconstituted humanized mice. *Biol. Open* **7**, doi:10.1242/bio.029785.
